# Associations between food intake and psychosomatic symptoms in 16-year-old adolescents

**DOI:** 10.1177/14034948241245770

**Published:** 2024-04-25

**Authors:** Maria Norburg Tell, Katarina Hedin, Mats Nilsson, Marie Golsäter, Hans Lingfors

**Affiliations:** 1 Futurum, Region Jönköping County, Jönköping, Sweden; 2 Department of Health, Medicine and Caring Sciences, Linköping University, Linköping, Sweden; 3 Department of Clinical Sciences in Malmö, Lund University, Malmö, Sweden; 4 Child Research Group, Department of Nursing, Jönköping University, Jönköping, Sweden

**Keywords:** Adolescents, healthy food intake, psychosomatic symptoms

## Abstract

**Aims:**

An increase in psychosomatic symptoms among adolescents has recently been reported. Few studies have examined the relation between food intake and psychosomatic symptoms. The aim was to study the association between food intake and overall psychosomatic burden and separate psychosomatic symptoms.

**Methods:**

In this cross-sectional study, we used data from 6248 girls and 7153 boys in south-east Sweden who turned 16 years of age during the academic years 2009/2010 to 2015/2016 and responded to a health questionnaire at the school health services. The association between overall healthy food intake and a low psychosomatic burden was calculated as odds ratios (95% confidence interval) and stratified for other lifestyle habits and gender.

**Results:**

Sixty-nine per cent of the boys and 35% of the girls had a low psychosomatic burden. There was a positive association between an overall healthy food intake and a low psychosomatic burden (*P*<0.0001), regardless of other lifestyle habits and gender. An overall healthy food intake was also positively associated with a lower frequency of the separate psychosomatic symptoms of concentration difficulties, sleep difficulties, a poor appetite or dizziness (*P*<0.0001).

**Conclusions:**

A healthy food intake seems to be associated with a low psychosomatic burden among adolescents. Further knowledge is needed to explore whether an improved food intake can reduce psychosomatic symptoms and enhance mental health.

## Background

Increasing rates of psychosomatic symptoms [1] have been reported among Nordic adolescents [2], especially among girls in Sweden [3]. Because of the increase in people suffering from psychosomatic symptoms, there is an urgent need for prevention and health promotion. Research indicates that diets such as the Mediterranean diet and the similar Nordic diet, which includes Nordic foods, not only have a preventive effect on cardiovascular diseases, but also positively impact brain function [4]. However, there is a limited number of studies exploring the relationship between food intake and psychosomatic symptoms [5, 6]. There is some evidence supporting the positive link between healthy food choices and good mental health [7–10] and less depression [11, 12]. More knowledge about the relations between food intake and psychosomatic symptoms as well as total psychosomatic burden is therefore of interest.

### Aims

The aim of this study among 16-year-old adolescents was to explore: (a) associations between overall food intake and psychosomatic burden; (b) associations between overall food intake and separate psychosomatic symptoms; and (c) associations between the intake of separate foods and separate psychosomatic symptoms.

## Methods

### Setting

According to the Swedish Education Act, adolescents should be offered a health visit at the school health services (SHSs) during their upper secondary school years [13]. The MyHealth questionnaire serves as the basis for a health dialogue to investigate and intervene in lifestyle habits during these health visits. Before the health visit, adolescents respond to the questionnaire, with support from the school nurse in the classroom. During the health visit, they have the opportunity to modify their answers. The questionnaire comprises 43 questions and 73 subquestions covering various aspects, including school situation, family and friends, physical activity, screen time, meals, food intake, sleep, psychosomatic symptoms, perceived health and self-image, as well as the use of tobacco, alcohol, and drugs and doping agents [14, 15].

The responses, along with anthropometric measurements of the adolescents taken at the health visit (height in metres to the nearest centimetre, without shoes, and weight in kilograms to the nearest 100 grams, in light clothing), are recorded in a database after the health dialogue.

#### MyHealth questionnaire: food intake and other lifestyle habits

The questions related to food intake concern the intake of fish, vegetables, fruit, mealtime beverages, butter/margarine as a sandwich spread, sandwich toppings, juice/chocolate drinks, sugar-sweetened beverages (SSBs), sweets/snacks and pastries. The questions were not primarily selected for research purposes, but serve as an underlay for discussions about food intake during lifestyle-oriented health dialogues at the SHSs, and were developed from a validated questionnaire for adults [14] and have been used in previous studies [15, 16].

The classification of the food questions was based on the food advice from the Swedish Food Agency [17]. The responses from unhealthy to healthy, were scored in points, in which a higher dietary score indicated an overall healthier food intake. The questions about food intake, the response alternatives and the points allocated for responses, presented as a dietary score, are given in Table I. The responses to the questions about physical activity and screen time were also scored. These scores were categorised into four levels, further distinguishing between high and low physical activity, as well as short and long screen time. Questions related to tobacco and alcohol use were categorised and further classified into no use and use. For additional details, including questions about physical activity, screen time, and tobacco and alcohol use see Supplemental file 1. For the classification of food intake and the other lifestyle habits please refer to Supplemental file 2 and for the graphic health curve tool used during health visits see Supplemental file 3.

**Table I.** Questions, response alternatives and scoring of food intake-related responses to MyHealth questionnaire items.

Questions: If you think of the past 7 days. . .	Response alternatives	Scoring of response alternatives	Classification of response alternatives
. . .how often did you have fish?	At least 3 times/week	3	Healthy food intake
	Twice/week	2	
	Once/week	1	Moderately healthy food intake
	Never	0	Unhealthy food intake
. . .how often did you have vegetables?	At least 3 times/day	3	Healthy
	About twice/day	3	
	About once/day	2	Moderately healthy
	5–6 Times/week	2	
	3–4 Times/week	1	
	1–2 Times/week	0	Unhealthy
	Never	0	
. . .how many fruits did you have?	At least 3 fruits/day	3	Healthy
	About 2 fruits/day	3	
	About 1 fruit/day	2	Moderately healthy
	About 3–4 fruits/week	1	
	About 1–2 fruits/week	0	Unhealthy
	No fruit	0	
. . .what mealtime beverages did you usually have?	Water	3	Healthy
	Milk (0.5% fat)	3	
	Milk (1.5% fat)	2	Moderately healthy
	Milk (3.0% fat)	1	Unhealthy
	Soda or juice	0	
. . .what butter/ margarine did you usually have as a sandwich spread?	None	1	Healthy
	30–40% Fat	3	
	60% Fat	1	Moderately healthy
	80% Fat	0	Unhealthy
. . .what sandwich toppings did you usually have?	None	2	Healthy
	Fruit or vegetables	3	
	Ham, turkey, egg, low-fat cheese, mackerel or caviar	3	
	Marmalade or honey	0	Unhealthy
	Sausage or high-fat cheese	1	
. . .how often did you have at least one glass of chocolate drink or juice?	Never	3	Healthy
	Once/week	3	
	2–3 Times/week	1	Moderately healthy
	4–5 Times/week	0	Unhealthy
	Almost every day	0	
	Several times/day	0	
. . .how often did you have at least one glass of soft drink or energy/sports drink (sugar-sweetened beverages)?	Never	3	Healthy
	Once/week	3	
	2–3 Times/week	1	Moderately healthy
	4–5 Times/week	0	Unhealthy
	Almost every day	0	
	Several times/day	0	
. . .how often did you have sweets or snacks (chocolate, ice cream, sweets, chips)?	Never	3	Healthy
	Once/week	3	
	2–3 Times/week	1	Moderately healthy
	4–5 Times/week	0	Unhealthy
	Almost every day	0	
	Several times/day	0	
. . .how often did you have cookies, biscuits, buns or cake (pastries)?	Never	3	Healthy
	Once/week	3	
	2–3 Times/week	1	Moderately healthy
	4–5 Times/week	0	Unhealthy
	Almost every day	0	
	Several times/day	0	

MyHealth questionnaire: psychosomatic symptoms.

The questionnaire comprises eight qualitative questions related to eight psychosomatic symptoms experienced by the adolescents over the past 6 months: concentration difficulties, sleeping difficulties, headache, stomach ache, tension, unhappiness, dizziness and poor appetite. For each question, there are five response alternatives: never, rarely, sometimes, often and always (see Table II). A psychosomatic symptom score was calculated by summing the eight questions. This score was then divided into four levels, classifying study persons as having either low or high psychosomatic burden (see Supplemental file 2).

**Table II.** Questions, response alternatives, and scoring of psychosomatic symptoms based on MyHealth questionnaire.

Questions: If you think of the past 6 months. . .	Response alternatives	Scoring of response alternatives	Boys *n* (%)	Girls *n* (%)
. . .have you had difficulty in concentrating? (answered by 7099 boys and 6206 girls)	Never	1	972 (14)	392 (6)
	Rarely	2	2785 (39)	1754 (28)
	Sometimes	3	2597 (37)	2856 (46)
	Often	4	636 (9)	1045 (17)
	Always	5	109 (2)	159 (3)
. . .have you had difficulty in sleeping? (answered by 7108 boys and 6214 girls)	Never	1	1807 (25)	1000 (16)
	Rarely	2	2490 (35)	1964 (32)
	Sometimes	3	1896 (27)	1990 (32)
	Often	4	736 (10)	1043 (17)
	Always	5	179 (3)	217 (3)
. . .have you suffered from headache? (answered by 7075 boys and 6200 girls)	Never	1	1982 (28)	786 (13)
	Rarely	2	3048 (43)	2051 (33)
	Sometimes	3	1522 (22)	1942 (31)
	Often	4	476 (7)	1215 (20)
	Always	5	47 (1)	206 (3)
. . .have you suffered from stomach ache? (answered by 7071 boys and 6196 girls)	Never	1	3253 (46)	1128 (18)
	Rarely	2	2663 (38)	2242 (36)
	Sometimes	3	867 (12)	1818 (29)
	Often	4	256 (4)	863 (14)
	Always	5	32 (0.45)	145 (2)
. . .have you felt tense? (answered by 7049 boys and 6 161 girls)	Never	1	2605 (37)	1274 (21)
	Rarely	2	2748 (39)	2117 (34)
	Sometimes	3	1305 (19)	1785 (29)
	Often	4	335 (5)	825 (13)
	Always	5	56 (1)	160 (3)
. . .have you had a poor appetite? (answered by 7078 boys and 6188 girls)	Never	1	3780 (53)	1970 (32)
	Rarely	2	2112 (30)	2150 (35)
	Sometimes	3	883 (12)	1432 (23)
	Often	4	253 (4)	526 (9)
	Always	5	50 (1)	110 (2)
. . .have you felt unhappy? (answered by 7085 boys and 6171 girls)	Never	1	2973 (42)	733 (12)
	Rarely	2	2752 (39)	2220 (36)
	Sometimes	3	1037 (15)	2241 (36)
	Often	4	278 (4)	832 (13)
	Always	5	45 (1)	145 (2)
. . .have you felt dizzy? (answered by 7033 boys and 6182 girls)	Never	1	3484 (50)	2130 (34)
	Rarely	2	2439 (35)	2171 (35)
	Sometimes	3	876 (12)	1346 (22)
	Often	4	199 (3)	473 (8)
	Always	5	35 (1)	62 (1)

Distribution of psychosomatic symptoms among 16-year-old boys (*n*=7153) and girls (*n*=6248) participating in a health dialogue at the school health services (SHSs) between 2009/2010 and 2015/2016.

*P*<0.0001 for gender for all items.

### Study population

The study population in this cross-sectional study consisted of 13,451 adolescents who turned 16 years of age during the academic years 2009/2010 to 2015/2016, and answered the MyHealth questionnaire and participated in health visits at the SHSs in Jönköping County, south-east Sweden [18]. The participating schools correspond to roughly half of the schools in the county. Data from study persons with missing responses on gender (*n*=50) were excluded from the analysis, resulting in completed questionnaires from 13,401 adolescents (6248 girls and 7153 boys).

### Data collection

Data from the questionnaires concerning psychosomatic symptoms, food intake, physical activity, screen time and tobacco and alcohol use, as well as anthropometric measurements, age and gender, were obtained from the above database.

### Statistical methods

Numbers and percentage were used for descriptive results. To examine the associations between food intake and psychosomatic symptoms, and between International Obesity Task Force standards body mass index (isoBMI; BMI classified into age and gender-specific groups) [19, 20] and psychosomatic symptoms, as well as differences between boys and girls, data were analysed with the chi² test. A *P* value less than 0.05 was considered statistically significant.

The odds ratio (OR) (with 95% confidence intervals (CIs)) for having a low psychosomatic burden among those with an overall healthy food intake and those with an overall unhealthy food intake were calculated, stratified by physical activity, screen time and tobacco and alcohol use.

The association between overall food intake (in four levels, see Table III) and the absence or presence of each psychosomatic symptom, from never to always (see Table II), was calculated, as was the association between each separate food intake (healthy vs. unhealthy) and the frequency of each psychosomatic symptom.

**Table III.** Distribution of overall food intake (dietary score) and psychosomatic burden (psychosomatic score), based on MyHealth questionnaire responses, among 16-year-old boys and girls (total *n*=13,401) participating in a health dialogue at the school health services (SHSs) between 2009/2010 and 2015/2016.

Item *n* = boys/girls	Categorisation of the item score	Boys *n*=7153 (%)	Girls *n*=6248 (%)	*P* value for gender
Overall food intake (dietary score) over the past 7 days^a^ *n*=7107/6224	Level 1 (25–30)	393 (6)	631 (10)	<0.0001
	Level 2 (19–24)	2377 (33)	2669 (43)	
	Level 3 (14–18)	2891 (41)	2127 (34)	
	Level 4 (0–13)	1446 (20)	797 (13)	
Psychosomatic burden (psychosomatic score) in the past 6 months^b^ *n*=6770/5935	Level 1 (8–13)	2407 (36)	711 (12)	<0.0001
	Level 2 (14–17)	2237 (33)	1389 (23)	
	Level 3 (18–21)	1379 (20)	1708 (29)	
	Level 4 (22–40)	747 (11)	2127 (36)	

a A high dietary score (level 1 and 2) suggests an overall healthy food intake (Supplemental file 2).

b A low psychosomatic score indicates a low psychosomatic burden (Supplemental file 2).

The psychometric properties of the psychosomatic problems (PSP) scale were tested in our data using Rasch analysis, which confirmed the results reported by Hagquist [21] (data not shown).

SAS/STAT 15.2 (SAS Institute Inc., Cary, NC, USA) was used for all analyses, except for the Rasch analyses, which were performed using RUMM2030 (Rumm Laboratory Pty. Ltd., Perth, Australia).

### Ethical considerations

This study was conducted according to the guidelines in the Declaration of Helsinki. All procedures involving human subjects were approved by the regional ethical review board of Linköping University, Linköping, Sweden (Dnr: 2018/19-31).

## Results

### Prevalence of psychosomatic burden

In our population, 53% of adolescents (69% of boys and 35% of girls) had a low psychosomatic burden (see Table II). The prevalence of eight separate psychosomatic symptoms experienced during the past 6 months, as reported in the questionnaire, is summarised in Table II. Forty-six per cent of the adolescents (39% of the boys and 53% of the girls) demonstrated an overall healthy food intake (see Table III). The distribution of separate food intakes and participants’ isoBMI has been presented elsewhere [16]. Notably, we found no significant association between isoBMI and the psychosomatic score. Therefore, we did not proceed any further with stratification by body weight.

### Associations between overall food intake and psychosomatic burden

We found a positive association (*P*<0.0001) between an overall healthy food intake and a low psychosomatic burden, regardless of the level of physical activity, screen time, tobacco or alcohol use, as well as gender (see Table IV).

**Table IV.** Association between food intake and psychosomatic burden, taking other lifestyle habits as well as gender into account, among 16-year-old boys (*n*=7153) and girls (*n*=6248) participating in a health dialogue at the school health services (SHSs) between 2009/2010 and 2015/2016.

Classification of lifestyle habits^a^	Classification of overall food intake	High psychosomatic burden *n* (%)		Low psychosomatic burden *n* (%)		Odds ratio for low psychosomatic burden for boys and girls with an overall healthy food intake (95% CI)	
		Boys	Girls	Boys	Girls	Boys	Girls
High physical activity	Healthy food intake	523 (26)	1390 (59)	1521 (74)	983 (41)	1.39 (1.22–1.58)	1.48 (1.30–1.68)
	Unhealthy food intake	907 (32)	1237 (68)	1899 (68)	593 (32)		
Low physical activity	Healthy food intake	89 (28)	318 (65)	232 (72)	172 (35)	1.86 (1.41–2.46)	1.44 (1.12–1.85)
	Unhealthy food intake	367 (42)	495 (73)	513 (58)	186 (27)		
Short screen time	Healthy food intake	434 (24)	1387 (57)	1355 (76)	1037 (43)	1.46 (1.27–1.68)	1.42 (1.25–1.61)
	Unhealthy food intake	721 (32)	1170 (66)	1537 (68)	615 (34)		
Long screen time	Healthy food intake	211 (32)	345 (70)	456 (68)	148 (30)	1.40 (1.16–1.70)	1.71 (1.32–2.21)
	Unhealthy food intake	647 (39)	641 (80)	997 (61)	161 (20)		
No tobacco use	Healthy food intake	482 (23)	1564 (57)	1647 (77)	1181 (43)	1.48 (1.31–1.68)	1.48 (1.32–1.67)
	Unhealthy food intake	935 (30)	1451 (66)	2155 (70)	739 (34)		
Tobacco use	Healthy food intake	189 (39)	288 (79)	291 (61)	76 (21)	1.46 (1.17–1.83)	1.48 (1.06–2.09)
	Unhealthy food intake	485 (49)	491 (85)	510 (51)	87 (15)		
No alcohol use	Healthy food intake	333 (21)	924 (53)	1231 (79)	815 (47)	1.60 (1.38–1.87)	1.48 (1.28–1.72)
	Unhealthy food intake	636 (30)	792 (63)	1467 (70)	472 (37)		
Alcohol use	Healthy food intake	346 (33)	934 (68)	703 (67)	444 (32)	1.34 (1.14–1.56)	1.53 (1.30–1.80)
	Unhealthy food intake	792 (40)	1150 (76)	1204 (60)	357 (24)		

a Classification of lifestyle habits (Supplemental files 1 and 2).

CI: confidence interval.

### Associations between overall food intake and separate psychosomatic symptoms

**Figure 1.**
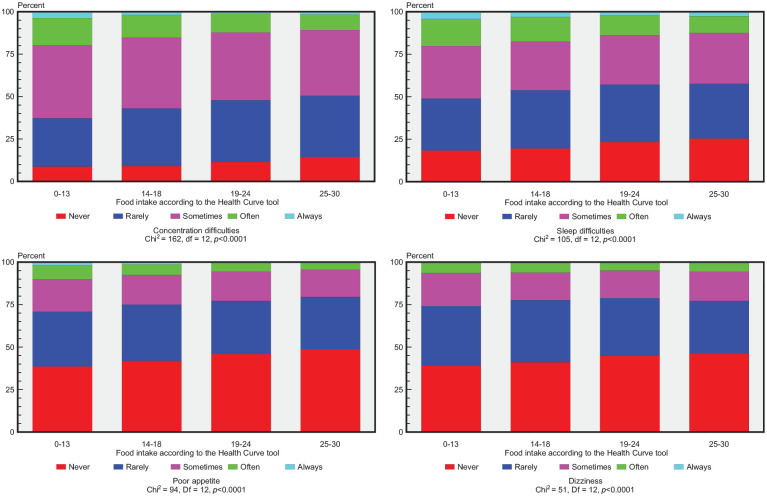
Distribution of psychosomatic symptoms in relation to overall food intake among 16-year-old adolescents participating in a health dialogue at the school health services (SHSs) between 2009/2010 and 2015/2016. A high dietary score suggests an overall healthy food intake (see Supplemental file 2).

There were statistically significant positive associations (*P*<0.0001) between reporting an overall healthy food intake and experiencing less frequent occurrences of the following separate psychosomatic symptoms: concentration difficulties, sleep difficulties and poor appetite or dizziness over the past 6 months (see Figure 1). In both the boys and the girls an overall healthy food intake was associated with lower frequencies of all eight psychosomatic symptoms (*P*<0.05), except for feeling unhappy among the boys.

### Associations between intake of separate foods and separate psychosomatic symptoms

When studying the distribution of the separate foods in relation to the separate psychosomatic symptoms, those who had a healthy intake of fish (twice or more/week vs. no fish) as well as those who had a low intake of sweets and snacks (once or less/week vs. almost every day or more) demonstrated a statistically significant lower frequency of all eight psychosomatic symptoms (*P*<0.05). Those who reported a healthy intake of vegetables (twice or more/day vs. once a week or less), a healthy intake of fruit (two or more fruits/day vs. no fruit), as well as a low intake of SSBs (one glass or less/week vs. almost every day or more often) had a lower frequency of concentration difficulties, sleeping difficulties or poor appetite (*P*<0.001) than those who had an unhealthy intake.

## Discussion

In this cross-sectional 7-year study among 13,401 16-year-old adolescents a positive association was found between an overall healthy food intake and a low psychosomatic burden, regardless of other lifestyle habits such as physical activity, screen time and tobacco and alcohol use, for both boys and girls. An overall healthy food intake, as well as all separate healthy food intakes, was related to a lower frequency of the separate psychosomatic symptom concentration difficulties, sleeping difficulties, poor appetite and dizziness. Furthermore, a high fish intake and a low intake of sweets and snacks was associated with a lower frequency of having a headache, having stomach ache, feeling tense and feeling unhappy.

### Prevalence of psychosomatic symptoms and psychosomatic burden

About half of the 16-year-olds in the present study had a high psychosomatic burden. The most frequently reported psychosomatic symptoms for the boys were concentration difficulties and sleeping difficulties. For the girls, common symptoms were headache, followed by concentration difficulties and sleeping difficulties. As in other studies, girls as a group had a higher prevalence of psychosomatic symptoms [3] but reported a healthier food intake than boys [15].

Aggregated local data from the medical record system in Region Jönköping County show that, from 2011 to 2021, 45% of adolescents turning 16 years of age had an International Classification of Diseases, 10th revised edition (ICD-10) diagnosis from a general practitioner (GP). Of these diagnoses, 24% indicated the prevalence of psychosomatic symptoms, 46% of which were related to stomach ache, 28% to headache, 9% to feeling tense, 7% to feeling dizzy, 5% to sleeping difficulties, 4% to feeling unhappy and 0.3% to a low appetite. When comparing our results with diagnostic codes from physicians in primary care, there is an indication that only a minority of 16-year-olds see a physician in primary care for their psychosomatic symptoms. The majority of psychosomatic symptoms in this age group were managed through self-care, through the SHSs or at special hospital units, with unknown distribution. The most common of the diagnostic codes related to psychosomatic symptoms used by physicians in primary care were related to having stomach ache and headache. The differences in distribution between our results and the local aggregated data confirm that adolescents seek care for various psychosomatic symptoms across different arenas of healthcare. This variation likely reflects the structure and responsibility of primary care in Sweden at the time of the study.

### Associations between overall food intake and psychosomatic burden

The results from the present study indicate an association between an overall healthy food intake and a low psychosomatic burden. The association between a healthy lifestyle and few psychosomatic symptoms is in line with an earlier study on children between 10 and 16 years of age [6]. However, that study focused on an overall healthy lifestyle, with the daily intake of fruit and vegetables as indicating a healthy food intake [6]. Other studies, in somewhat older populations than ours, point in the same direction [5, 22, 23].

A healthy food intake may contribute to a low psychosomatic burden or may just be a marker for a low psychosomatic burden, and may reflect something else that might be associated with a low psychosomatic burden among adolescents. One possible confounder could have been bodyweight [24]. However, in this study there was no association between bodyweight and psychosomatic score. As discussed previously [4, 25], there is some support for the hypothesis that food intake per se might influence psychosomatic symptoms. Gender differences in the prevalence of psychosomatic burden cannot be solely explained by differences in food intake. These results raise questions about the underlying mechanisms driving associations between food intake and psychosomatic burden, and whether gender plays a role. There is a need for further studies with a broad range of study designs to gain a better understanding of these associations. For future studies, there is also a need for more data, such as socioeconomic status, which may be an underlying reason for the links between food intake and psychosomatic burden.

### Strengths and weaknesses

Our study possesses several strengths, as well as some limitations. The inclusion of a substantial number of 16-year-olds contributes to the robustness of our findings. The large proportion of participating municipalities that used the questionnaire enhances its representativeness for the county. Previous studies have concluded that Jönköping County is fairly representative of the whole country [26]. However, the proportion of inhabitants with higher education in Jönköping County is slightly lower compared with the rest of Sweden. There is also a lower proportion of inhabitants with a foreign background [27].

The study design itself is another strength, including comparison between an overall food intake score and a psychosomatic score and also separate psychosomatic symptoms, as well as comparison between intakes of separate food items, and separate psychosomatic symptoms. Criticism has been raised in the literature regarding the method of combining severe and trivial psychological symptoms in a score [28]. Our study deals with this risk by performing analyses also at a separate psychosomatic symptom level. The method used indicated that food intake is crude and may not fully capture the nuances of dietary habits. It is also a limitation that the food intake is self-reported, as well as it is a drawback that the scores are not validated. Data collection through anonymously answered questionnaires carries the risk of misunderstandings that can lead to misreporting and non-response. However, this risk is reduced in the present study because the questionnaire was used as a basis for a dialogue at the SHSs. This context gave the included adolescents the opportunity to adjust their answers and complete all the questions at the health visit, which may have increased the validity.

It is possible that lack of anonymity may have affected the truthfulness of the responses. However, the importance of adolescents feeling understood by the school nurse has been highlighted in an interview study within the same context [18]. It is therefore likely that the adolescents gave truthful answers in order to receive the right support from the school nurse. The absence of data for controlling analyses regarding various factors, including socioeconomic status represents a limitation. These variables could potentially impact both psychosomatic symptoms and food intake.

### Clinical implications

Swedish adolescents have shown an increase in psychosomatic symptoms over time [3] and there is therefore an urgent need to find ways to address this matter. At the same time, few adolescents report a healthy food intake [29]. The results from this study regarding the association between a healthy food intake and low psychosomatic burden is therefore clinically relevant. To our knowledge, there is limited research on the relation between a healthy food intake and psychosomatic symptoms in 16-year-olds. Food advice supported by arguments about a possible risk for future disease may not be motivational enough for a healthy lifestyle behaviour during adolescence. It is plausible that other factors, with short-term effects, play a more influential role. It should be borne in mind that adolescence is a period in life when the ability to plan and the understanding of consequences are still developing [30]. Knowledge about associations between food intake and psychosomatic symptoms may be beneficial as it may serve as a motivational argument beyond long-term risks. This understanding should be considered when designing interventions for healthy food intake or enhanced mental health among adolescents. Lifestyle behaviour is a factor that an individual has the possibility to control to some extent. Consequently, there is a need for deeper knowledge about whether a change from a less healthy to a healthier diet can affect mental health.

## Conclusions

A healthy food intake seems to be associated with a low psychosomatic burden among adolescents. Further knowledge is needed to explore whether an improved food intake can reduce psychosomatic symptoms and enhance mental health.

## Supplemental Material

sj-docx-1-sjp-10.1177_14034948241245770 – Supplemental material for Associations between food intake and psychosomatic symptoms in 16-year-old adolescents Supplemental material, sj-docx-1-sjp-10.1177_14034948241245770 for Associations between food intake and psychosomatic symptoms in 16-year-old adolescents by Maria Norburg Tell, Katarina Hedin, Mats Nilsson, Marie Golsäter and Hans Lingfors in Scandinavian Journal of Public Health

sj-docx-2-sjp-10.1177_14034948241245770 – Supplemental material for Associations between food intake and psychosomatic symptoms in 16-year-old adolescents Supplemental material, sj-docx-2-sjp-10.1177_14034948241245770 for Associations between food intake and psychosomatic symptoms in 16-year-old adolescents by Maria Norburg Tell, Katarina Hedin, Mats Nilsson, Marie Golsäter and Hans Lingfors in Scandinavian Journal of Public Health

sj-jpg-1-sjp-10.1177_14034948241245770 – Supplemental material for Associations between food intake and psychosomatic symptoms in 16-year-old adolescents Supplemental material, sj-jpg-1-sjp-10.1177_14034948241245770 for Associations between food intake and psychosomatic symptoms in 16-year-old adolescents by Maria Norburg Tell, Katarina Hedin, Mats Nilsson, Marie Golsäter and Hans Lingfors in Scandinavian Journal of Public Health
