# Supplementary material for: Associations between food intake and psychosomatic symptoms in 16-year-old adolescents
Source: Scand J Public Health. 2024 Apr 25;53(4):367–75. doi: 10.1177/14034948241245770 (PMC12048730; doi:10.1177/14034948241245770)
Supplement: sj-docx-1-sjp-10.1177_14034948241245770 – Supplemental material for Associations between food intake and psychosomatic symptoms in 16-year-old adolescents [file sj-docx-1-sjp-10.1177_14034948241245770.docx]

Supplement 1

| **Questions** | **Response alternatives** | **Scoring of response alternatives** |
| --- | --- | --- |
| **Thinking about the last 7 days …,**  how **physically active** have you been?  PHYSICAL ACTIVITY (PA) means all activity that warms you up (e.g. walking, cycling) or makes you out of breath (e.g. school sports, jogging, gymnastics, cycling, swimming, ball games or dance). | **Time spent in physical activity, in  hours each day,  over the last 7 days** | **Physical activity points** |
|  | <0.5 | 0 |
|  | 0.5 | 2 |
|  | 1 | 4 |
|  | 1.5 | 5 |
|  | 2 |  |
|  | 3 |  |
|  | >4 |  |
| **Thinking about the last 7 days …,**  how **physically inactive** have you been?  PHYSICAL INACTIVITY in this case means SCREEN TIME when you were sitting still at a PC/mobile phone or watching TV or a film or playing a video game during your free time. | **Time spent in viewing screens, in  hours each day,  over the last 7 days** | **Screen time points** |
|  | <0.5 | 0 |
|  | 0.5 | 0.5 |
|  | 1 | 1 |
|  | 1.5 | 1.5 |
|  | 2 | 2 |
|  | 3 | 3 |
|  | >4 | 4 |
| **Do you smoke?** | **Response alternatives** | **Tobacco points** |
|  | No | 3 |
|  | I smoke cigarettes less than once a week | 0 |
|  | I smoke cigarettes at least once a week, but not every day |  |
|  | I smoke cigarettes every day |  |
|  | I smoke a shisha less than once a week |  |
|  | I smoke a shisha at least once a week, but not every day |  |
|  | I smoke a shisha every day |  |
| **Do you use snuff?** | No | 3 |
|  | Yes, I use snuff less than once a week | 0 |
|  | Yes, I use snuff at least once a week, but not every day |  |
|  | Yes, I use snuff every day |  |
| **Do you drink alcohol?**  ALCOHOL here means  drinks like beer, strong cider, wine, alcopops or spirits. | **Response alternatives** | **Alcohol points** |
|  | No | 6 |
|  | Yes, no more than 2–3 times a year | 2 |
|  | Yes, between once a month and every other month | 1 |
|  | Yes, every week | 0 |
