# Supplementary material for: Associations between food intake and psychosomatic symptoms in 16-year-old adolescents
Source: Scand J Public Health. 2024 Apr 25;53(4):367–75. doi: 10.1177/14034948241245770 (PMC12048730; doi:10.1177/14034948241245770)
Supplement: sj-docx-2-sjp-10.1177_14034948241245770 – Supplemental material for Associations between food intake and psychosomatic symptoms in 16-year-old adolescents [file sj-docx-2-sjp-10.1177_14034948241245770.docx]

Supplement 2

| **Item** | Score | Categorization | Classification |
| --- | --- | --- | --- |
| **Overall food intake *** (Dietary score) | 25–30 19–24 | Level 1  Level 2 | Healthy food intake |
|  | 14–18 0–13 | Level 3 Level 4 | Unhealthy food intake |
| **Physical activity ***  (Physical activity score) | 25–35 17–24 | Level 1 Level 2 | High physical activity |
|  | 12–16 0–11 | Level 3 Level 4 | Low physical activity |
| **Screen time ***  (Screen time score) | 0–14 15–20 | Level 1  Level 2 | Short screen time |
|  | 21–25 >25 | Level 3 Level 4 | Long screen time |
| **Tobacco use ***  (Tobacco score) | 6 | Level 1 | No use of tobacco |
|  | 0–3 | Level 4 | Use of tobacco |
| **Alcohol use ***  (Alcohol score) | 0–2 | Level 1 | No use of alcohol |
|  | 6 | Level 4 | Use of alcohol |
| **Psychosomatic burden**  (Psychosomatic score) | 8–13 14–17 | Level 1  Level 2 | Low psychosomatic burden |
|  | 18–21 22–40 | Level 3 Level 4 | High psychosomatic burden |

*Level regarding lifestyle habits refers to the classification in the Health Curve tool (see Supplement 3).
