# Supplementary figures and images for: Associations between food intake and psychosomatic symptoms in 16-year-old adolescents
Source: Scand J Public Health. 2024 Apr 25;53(4):367–75. doi: 10.1177/14034948241245770 (PMC12048730; doi:10.1177/14034948241245770)

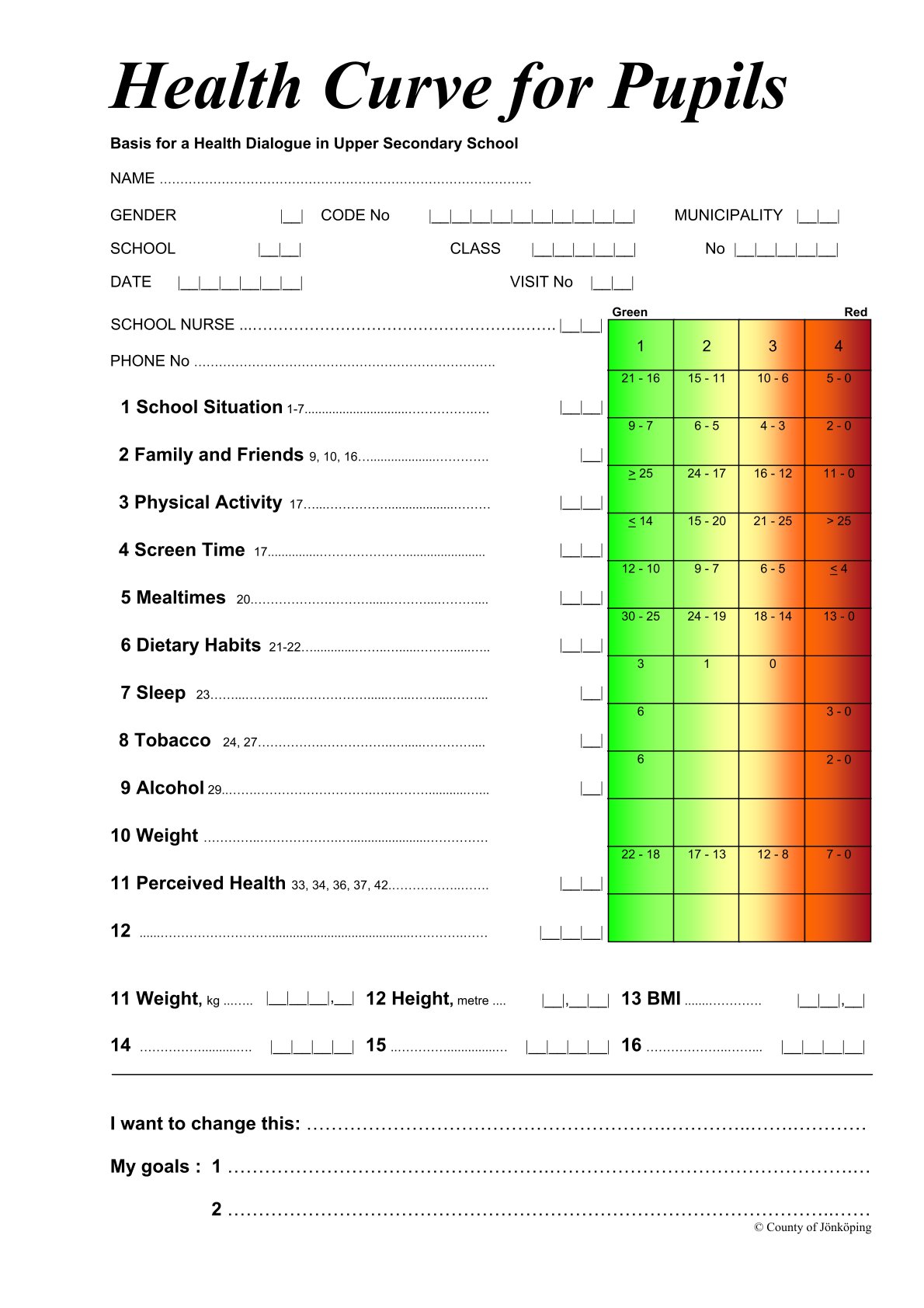

Supplement: sj-jpg-1-sjp-10.1177_14034948241245770 – Supplemental material for Associations between food intake and psychosomatic symptoms in 16-year-old adolescents [file sj-jpg-1-sjp-10.1177_14034948241245770.jpg]
